# Molecular characterization of clinical responses to PD‐1/PD‐L1 inhibitors in non‐small cell lung cancer: Predictive value of multidimensional immunomarker detection for the efficacy of PD‐1 inhibitors in Chinese patients

**DOI:** 10.1111/1759-7714.13078

**Published:** 2019-04-23

**Authors:** Peng Song, Xiaoxia Cui, Li Bai, Xiangdong Zhou, Xiaoli Zhu, Jian Zhang, Faguang Jin, Jianping Zhao, Chengzhi Zhou, Yanbin Zhou, Xiaoju Zhang, Kai Wang, Qi Wang, Yao Yu, Xiaoyu Zhang, Chunxue Bai, Li Zhang

**Affiliations:** ^1^ Department of Respiratory Medicine, Peking Union Medical College Hospital Peking Union Medical College & Chinese Academy of Medical Sciences Beijing China; ^2^ Department of Respiratory Disease, Xinqiao Hospital Third Military Medical University Chongqing China; ^3^ Department of Respiratory Medicine Southwest Hospital, Third Military Medical University Chongqing China; ^4^ Department of Respiratory, Zhongda Hospital Southeast University Nanjing China; ^5^ Department of Respiratory Medicine, Xijing Hospital Air Force Medical University Xi'an China; ^6^ Department of Respiratory Medicine Tangdu Hospital, Air Force Medical University Xi'an China; ^7^ Divsion of Respiratory Diseases, Department of Internal Medicine Tongji Hospital of Tongji Medical College of Huazhong University of Science and Technology Wuhan China; ^8^ State Key Laboratory of Respiratory Disease, National Clinical Research Center for Respiratory Disease, Guangzhou Institutes of Respiratory Disease The First Affiliated Hospital of Guangzhou Medical University Guangzhou China; ^9^ Department of Respiratory Medicine Sun Yat‐sen University First Affiliated Hospital Guangzhou China; ^10^ Department of Pulmonary Medicine He'nan Provincial People's Hospital Zhengzhou China; ^11^ Department of Pulmonary Medicine The Second Affiliated Hospital, Zhejiang University Hangzhou China; ^12^ Department of Pulmonary Medicine The Second Hospital of Dalian Medical University Dalian China; ^13^ GloriousMed Technology Co.,Ltd. Shanghai China; ^14^ Department of Respiratory Medicine Zhongshan Hospital, Fudan University Shanghai China

**Keywords:** Multi‐omics, NSCLC, PD‐1/PD‐L1 inhibitor, predictive biomarker

## Abstract

According to multiple studies, the objective response rate of PD‐1/PD‐L1 inhibitors in the second‐line treatment of unscreened non‐small cell lung cancer (NSCLC) is only approximately 20%. Predictive biomarkers of treatment efficacies are still under investigation. In selected NSCLC patients with PD‐L1 expression ≥ 50%, the response rate of pembrolizumab in first‐line treatment can reach 44.8%. Moreover, patients with a higher tumor mutation burden (TMB) tend to achieve a better response with nivolumab. Besides PD‐L1 expression and TMB, taking all these indicators into consideration would hypothetically maximize the clinical response in a specific subgroup of patients. Our study aims to accumulate large and complete samples and clinical data to verify the biomarkers and their cutoff values related to the efficacy of PD‐1/PD‐L1 inhibitors in Chinese NSCLC patients, and to construct a comprehensive predictive model by combining multi‐omics data with contemporary machine learning techniques. NSCLC patients administered treatment of anti‐PD‐1/PD‐L1 antibodies or a combination with other drugs have been enrolled. The estimated enrollment is 250 participants. A sophisticated predictive model of immunotherapy response in the Chinese population has not yet been developed. It is clinically and practically imperative to comprehensively evaluate the possible indicators of Chinese NSCLC patients through multiple test platforms, such as next generation sequencing, PCR, or immunohistochemistry. This study is registered in the Chinese Clinical Trial Registry (ChiCTR1900021395).

## Introduction

Lung cancer ranks first worldwide in terms of morbidity and mortality among all malignant tumors. In China in 2015, approximately 4.292 million new cases and 2.814 million deaths from cancer were estimated, including approximately 700 000 new lung cancer cases, of which non‐small cell lung cancer (NSCLC) accounts for approximately 85%.^1^ With 14 lung cancer targeting drugs approved by the Food and Drug Administration/National Medical Products Administration, the mode of diagnosis and treatment of lung cancer has developed from the pathology‐based chemo‐monotherapies of the past to the current molecular‐based targeted drugs, which act with higher specificity, a faster response, and lower toxicity.^2^, ^3^, ^4^ EGFR‐tyrosine kinase inhibitors (TKIs, e.g. gefitinib, erlotinib, afatinib, etc.) show both longer median progression‐free survival (mPFS) and objective response rates (ORRs) in first‐line or second‐line treatment of lung cancer. For example, in a phase III study of gefitinib used for the first‐line treatment of NSCLC in East Asia, the one‐year PFS rates were 24.9% with gefitinib and 6.7% with carboplatin‐paclitaxel. In the *EGFR* mutation‐positive subgroup, patients administered gefitinib had longer PFS than others administered chemotherapy (hazard ratio [HR] for progression 0.48, 95% confidence interval [CI] 0.36–0.64; *P* < 0.001; mPFS, 9.5 vs. 6.3 months), but in the *EGFR* mutation‐negative subgroup, patients administered gefitinib had shorter PFS than others administered chemotherapy (HR 2.85, 95% CI 2.05–3.98; *P* < 0.001; mPFS, 5.5 vs. 1.5 months).^5^, ^6^

Recently, research in cancer immunotherapy has been thriving. Inhibition of the immunological checkpoint pathway represented by PD‐1/PD‐L1 in cancer immunotherapy is considered one of the most promising treatment methods. There are currently three PD‐1/PD‐L1 inhibitors approved by the FDA for NSCLC, namely nivolumab,^7^, ^8^ pembrolizumab,^9^ and atezolizumab.^10^ Intriguingly, the clinical application of PD‐1/PD‐L1 inhibitors has been met with controversy. Multiple studies have revealed that the response rate of PD‐1/PD‐L1 inhibitors in second‐line treatment of unscreened NSCLC is merely approximately 20%.^7^, ^8^, ^9^, ^10^, ^11^ Meanwhile, in selected NSCLC patients with PD‐L1 expression ≥ 50%, the response rate of pembrolizumab in first‐line treatment can reach 44.8%.^9^ The CHECKMATE‐026 study focusing on nivolumab in NSCLC first‐line treatment showed no significant improvement in the nivolumab compared to the chemotherapy cohort (PFS 4.2 vs. 5.9 months; OS 14.4 vs. 13.2 months). But retrospective analysis of subgroups according to tumor mutation burden (TMB) found that patients with higher TMB tend to achieve a better response and superior PFS from nivolumab (nivolumab vs. chemotherapy: ORR 47% vs. 28%, mPFS, 9.7 vs. 5.8 months).^12^ In CHECKMATE‐227, among the subgroup patients with high TMBs (≥ 10 mutations/Mbp), patients administered nivolumab plus ipilimumab had longer PFS than those administered chemotherapy.^13^

These results, among others, suggest that not all NSCLC patients benefit from PD‐1/PD‐L1 inhibitors. A better clinical response might be achieved with parallel tests and analyses concerning other biomarkers, including PD‐L1 expression,^14^, ^15^ TMB,^16^, ^17^ metagenomic sequencing of intestinal microbiota, and certain somatic mutations. Taking all of these indicators into consideration would hypothetically maximize the clinical response of a specific subgroup of patients. In the CHECKMATE‐026 trial, patients administered nivolumab with high TMB and PD‐L1 expression ≥ 50% had a higher response rate than patients with high TMB or PD‐L1 expression ≥ 50% administered other treatment (75% vs. 32% vs. 34%).^12^ In KEYNOTE‐028, a study of pembrolizumab treatment across 20 cancer types, higher T‐cell‐inflamed gene‐expression profile (GEP), PD‐L1 expression, and/or TMB were associated with higher response rates and longer PFS. The subgroup of patients with tumors that had both high TMB and high inflammatory markers (GEP or PD‐L1) had the highest likelihood of response.^18^

The expression levels of some proliferation‐associated genes are associated with response to immune checkpoint inhibitors in NSCLC.^19^ Additionally, *JAK1/2* mutations are associated with resistance to anti‐PD‐1/PD‐L1 antibodies^20^ and *MDM2/MDM4* and *EGFR* alterations may correlate with hyperprogression.^21^, ^22^ The National Comprehensive Cancer Network guideline for NSCLC recommends PD‐1 inhibitors as first‐line treatment for patients with PD‐L1 expression ≥ 50% without *EGFR* or *ALK* mutations, and pembrolizumab for patients with PD‐L1 expression ≥ 1% as second‐line treatment. In January 2018, multiple studies confirmed that the composition of specific intestinal microbiota in Western populations significantly prolonged PFS in patients treated with PD‐1/PD‐L1 inhibitors.^23^, ^24^ However, a sophisticated predictive model of immunotherapy response in the Chinese population has not yet been developed. It is clinically and practically imperative to comprehensively evaluate the possible indicators of Chinese NSCLC patients through multiple test platforms, such as next generation sequencing (NGS), PCR, or immunohistochemistry (IHC).

## Methods

### Objectives

The main purposes of this prospective observational study are to accumulate large and complete samples and clinical data to verify the biomarkers and their cutoff values related to the efficacy of PD‐1/PD‐L1 inhibitors in Chinese NSCLC patients, and to construct a comprehensive predictive model from the data. The secondary purposes are to explore the relationship between numbers and/or frequencies of certain genetic variations (single nucleotide variations, insertions and deletions, copy number variations) and immunotherapy response, and to verify the influence of known DNA repair‐related genes on immunotherapy resistance and/or hyperprogressive disease. This study will also focus on the application of liquid biopsy, especially the mutation spectrum and TMB in circulating tumor DNA (ctDNA) for discriminating pseudoprogression and predicting resistance or progression of immunotherapy. An evaluation of intestinal microbiota metagenomic sequencing, based on the efficacy of PD‐1/PD‐L1 inhibitors and changes in intestinal microecology in Chinese tumor patients, will also be conducted in conditional research centers.

### Eligibility criteria

The estimated enrollment is 250 participants. Enrollment is limited to patients: aged 18–75 years old; with histologically or cytologically confirmed metastatic or local advanced NSCLC; without any *EGFR* or *ALK* genetic alteration (common or uncommon); administered anti‐PD‐1/PD‐L1 antibodies or a combination with other drugs; with available tumor tissue samples within two years before treatment or undergoing biopsies; with no less than 15 formalin‐fixed paraffin‐embedded samples; with available peripheral blood samples obtained within one week before treatment, and six weeks, three months and six months after treatment, and at the time point of withdrawal (10 mL each sample); at least one measurable lesion according to Response Evaluation Criteria in Solid Tumors (RECIST) version 1.1; Eastern Cooperative Oncology Group performance status (ECOG PS) score of 0 or 1; life expectancy of at least 12 weeks; and with adequate organ function and hematopoiesis. Subjects with a history of hematologic or primary solid tumor malignancy within five years prior to the first study treatment are excluded. The ethics committees at Zhongshan Hospital of Fudan University and each institution approved this study and written informed consent will be obtained from each patient.

### Study design and treatment plan

**Figure 1:**
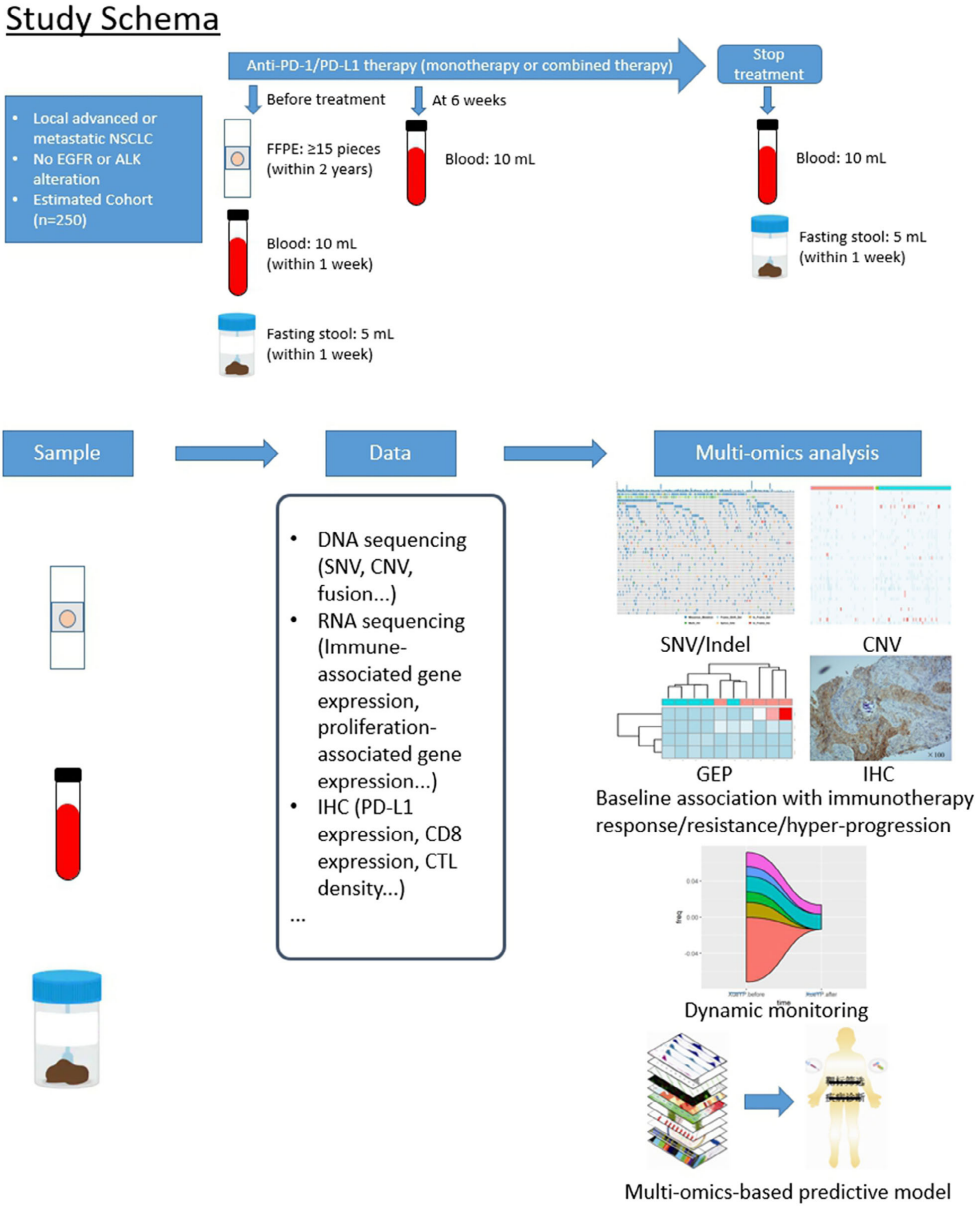
Study schema of ChiCTR1900021395. CNV, copy number variation, FFPE, formalin‐fixed, paraffin‐embedded; GEP, gene‐expression profile; IHC, immunohistochemistry; Indel, insertions and deletions; NSCLC, non‐small cell lung cancer; SNV, single nucleotide variations.

This study is a prospective, single‐arm, observational study. Primary tumor tissues, serial liquid biopsies, and stool samples will be obtained from patients with advanced NSCLC, with no *EGFR* or *ALK* genomic tumor aberrations treated with PD‐1 or PD‐L1 inhibitors (Fig 1).

The primary efficacy endpoint is to identify a novel predictive model for ORR and PFS after treatment via a comprehensive association analysis using multi‐omics data. For baseline evaluation, formalin‐fixed paraffin‐embedded tumor tissues, 10 mL of peripheral blood, and 5 mL of fasting stool in the morning will be collected from registered patients before treatment. Six weeks, three months and six months after the initiation of PD‐1/PD‐L1 treatment, 10 mL of peripheral blood will be sampled from patients that are still involved. If the treatment needs to be discontinued as the result of disease progression or intolerable toxicity, 10 mL of peripheral blood and 5 mL of fasting stool in the morning will be collected at the point of patient withdrawal. Consents will be completed and archived at each surveillance and sample collection. All data should be available for analysis by the end of 2019 (Table 1).

**Table 1:** Project flow chart

Stage	Baseline	Supervision period			
	V0	V1	V2	V3	Vx
Item	‐1–0 weeks	6 weeks ± 3 days	3 months ± 3 days	6 months ± 3 days	Discontinued
General information	×				
Pathology Report	×				
Eligibility criteria	×				
Informed consent	×				
FFPE samples	×				
Blood samples	×	×	×	×	×
Stool samples	×				×
CRF	×	×	×	×	×
Adverse reactions	×	×	×	×	×

CRF, case report form; FFPE, formalin‐fixed paraffin‐embedded.

### Expected results

First, using comprehensive analyses of multi‐omics data, novel predictive markers or models associated with the response to anti‐PD‐1/PD‐L1 antibody treatment should be revealed. Second, immunotherapy resistance‐associated mutations will be identified with ctDNA analysis. Finally, a strategy for the rapid determination of therapeutic outcomes by evaluating dynamic changes in ctDNA will be obtained.

### Analytical methods

**Figure 2:**
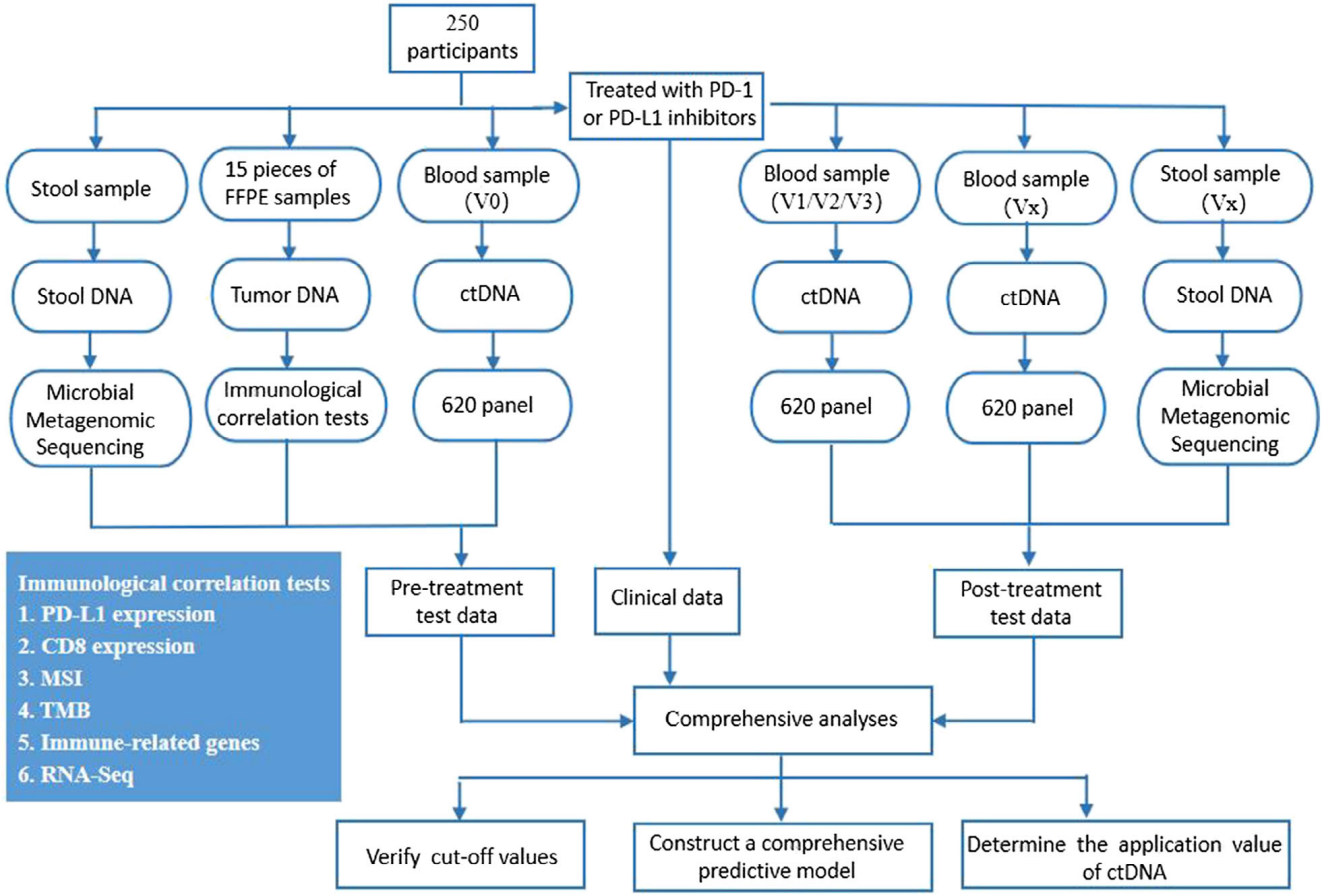
Technical roadmap of the study. CtDNA, circulating tumor DNA; FFPE, formalin‐fixed paraffin‐embedded; MSI, microsatellite instability; TMB, tumor mutation burden.

Comprehensive analyses will be performed as follows: (i) somatic mutation analysis with tumor DNA using whole exon sequencing; (ii) somatic mutation analysis with ctDNA using NGS for 620 cancer‐associated genes; (iii) gene expression profiling analysis using RNA‐Seq; (iv) microsatellite instability analysis using PCR; and (v) PD‐L1 and CD8 expression analysis using immunohistochemical staining (Fig 2). Statistical association analysis between the acquired data and clinical efficacy will be performed to identify candidates that achieve a good outcome after anti‐PD‐1 or PD‐L1 treatment.

All of the data obtained from six layers will be statistically analyzed to develop a robust clinical predictive model of immunotherapy using the following framework. We will identify the biomarkers for predicting patient response to PD‐1 or PD‐L1 inhibitors from each layer. We will then combine multi‐omics data with contemporary machine learning techniques to develop predictive models. We intend to focus on drug resistance mechanisms; in particular, we will explore acquired mutations during treatment via ctDNA analysis. This will hopefully allow us to understand the mechanisms of resistance and highlight the potential mutations that can be targeted by novel therapeutics. Furthermore, we will investigate whether the effectiveness of immunotherapy can be predicted based on changes in ctDNA levels. We will compare the timing and magnitude of changes in ctDNA levels and radiographic tumor size measurements longitudinally during treatment. We will examine whether patients with downtrending ctDNA levels are more likely to gain a prolonged treatment benefit. We will also examine whether such patients have improved PFS. Furthermore, we seek to determine if baseline and early therapy measurements of ctDNA can differentiate pseudoprogression from true progression in patients treated with immunotherapy.

### Feasibility analysis

The study design has been validated in numerous clinical trials. Multiple platforms will be introduced, such as NSG, Sanger sequencing, metagenomic sequencing, PCR, RNA‐seq, and IHC. TMB will be detected by NGS, and immune‐related gene expression profiles by the RNA‐seq platform. PD‐L1 and CD8 expression will be detected by IHC. The detection of thousands of samples has validated these platforms. By means of these platforms, DNA and RNA in different types of samples will be detected and we can then obtain adequate information. We will analyze valuable information in the view of genomics, RNomics, and proteomics and screen biomarkers to predict therapeutic effects of immunotherapy. Moreover, testing samples are available. CtDNA from peripheral blood and microbiome DNA from excrement will be obtained and detected. Finally, the medical records of each patient will be reviewed and a paper case report form completed by well‐trained study physicians, which will be updated manually. Professional data entry staff will manually review the form and transcribe clinical and sequencing data into an electronic data capture system specifically designed for this study.

## Discussion

The primary goal of this study is to establish a multicenter cohort of NSCLC patients in China. This result will complement current understanding of the use of PD‐1/PD‐L1 inhibitors to predict biomarkers in advanced Chinese NSCLC patients and explore the relevant influencing factors. Through long‐term follow‐up of patients, we can further improve real‐world multicenter data of the efficacy of advanced NSCLC immunotherapy in China.^25^

This multicenter prospective study will deepen the mining of multiple biomarkers, establish a comprehensive forecasting model, and predict the sensitivity of treatment in multiple dimensions. We believe that biomarkers indicating the efficacy of PD‐1 can be integrated into a mathematical model, which will greatly improve the predictive value of immunotherapy.

However, this study does have potential limitations, such as the inconsistencies and incompleteness of clinical practice data sources; diagnostic bias among different institutions; the fact that blinding is not performed in a real‐world study; and intervention by external clinical trial participants. Research methods with higher statistical power will help to reduce these biases.

## Disclosure

No authors report any conflict of interest.
